# Population-Based Cancer Prevention Education Intervention Through mHealth: A Randomized Controlled Trial

**DOI:** 10.1007/s10916-023-02026-y

**Published:** 2024-01-09

**Authors:** Carolina Espina, Ariadna Feliu, Albert González Vingut, Theresa Liddle, Celia Jimenez-Garcia, Inmaculada Olaya-Caro, Luis Ángel Perula-De-Torres

**Affiliations:** 1 International Agency for Research on Cancer (IARC/WHO), Environment and Lifestyle Epidemiology Branch, 25 avenue Tony Garnier CS 90627, 69366 Lyon CEDEX 07, France; 2 Health Emergencies Center 061, CRM e I+D+I Salud Responde, Consejería de Salud y Consumo, Junta de Andalucía, Seville, Spain; 3 Health Emergencies Center 061, Salud Responde, Consejería de Salud y Consumo, Junta de Andalucía, Jaén, Spain; 4 Maimonides Institute of biomedical Research of Cordoba (IMIBIC)/University of Cordoba, Córdoba, Spain; 5 Health District of Cordoba-Guadalquivir, Córdoba, Spain; 6 Programa de Actividades de Promoción y Prevención de la Salud (PAPPS-semFYC), Barcelona, Spain; 7 Chronicity, Primary Care and Health Promotion Research Network (RICAPPS), Cooperative Research Networks Oriented to Health Results (RICORS), Carlos III Health Institute, Madrid, Spain

**Keywords:** mHealth, Cancer prevention, European Code Against Cancer, Digital health, Implementation research, Mobile phone, Primary prevention, Cancer screening

## Abstract

Despite the high potential of mHealth-related educational interventions to reach large segments of the population, implementation and adoption of such interventions may be challenging. The objective of this study was to gather knowledge on the feasibility of a future cancer prevention education intervention based on the European Code Against Cancer (ECAC), using a population-based mHealth implementation strategy. A type-2 hybrid effectiveness-implementation study was conducted in a sample of the Spanish general population to assess adoption, fidelity, appropriateness, and acceptability of an intervention to disseminate cancer prevention messages, and willingness to consult further digital information. Participation rates, sociodemographic data, mHealth use patterns and implementation outcomes were calculated. Receiving cancer prevention messages through mHealth is acceptable, appropriate (frequency, timing, understandability and perceived usefulness) and feasible. mHealth users reported high access to the Internet through different devices, high ability and confidence to browse a website, and high willingness to receive cancer prevention messages in the phone, despite low participation rates in comparison to the initial positive response rates. Although adoption of the intervention was high, post-intervention fidelity was seriously hampered by the disruptions caused by the Covid-19 pandemic, which may have affected recall bias. In the context of the Europe’s Beating Cancer Plan to increase knowledge about cancer prevention across the European Union, this study contributes to inform the design of future interventions using mHealth at large scale, to ensure a broad coverage and adoption of cancer prevention messages as those promoted by the ECAC.

*Trial Registration:* ClinicalTrials.gov from the U.S. National Library of Medicine*,* NCT05992792*.* Registered 15 August 2023 - Retrospectively registered https://clinicaltrials.gov/study/NCT05992792?cond=Cancer&term=NCT05992792&rank=1.

## Background

Cancer is currently the second leading cause of death worldwide and the projections estimate that, not only cancer deaths will likely surpass other diseases and become the leading cause of death globally, but cancer will also pose a dramatic economic burden as populations age. In 2020, 282 421 new cancer cases were diagnosed in both sexes in Spain and 113 054 people died because of cancer. Colorectal, prostate and breast cancers were the most common cancers, while prostate, colorectal and lung cancers were the most lethal ones [1]. It is well established that at least 40% of cancers are attributable to modifiable risk factors such as tobacco and alcohol consumption, overweight, or sun exposure, therefore, primary prevention would be possible by avoiding or reducing exposure to certain risk factors and modifying unhealthy behaviours [2, 3]. Likewise, secondary prevention through cancer screening to detect certain types of cancer early, followed by effective treatment, are the main tools to avoid deaths from cancer [4]. Thus, prevention offers the most cost-effective cancer control strategy in the long term and, therefore, great potential for public health [5].

Health education and health promotion aim to increase individual and societal awareness of health and its determinants, as well as to develop and strengthen the health empowerment of the individual. From this perspective, cancer prevention education would improve the knowledge about established cancer risk factors, unhealthy lifestyles, and evidence-based interventions towards reducing the cancer burden. Consequently this may affect individuals' willingness to adopt and maintain healthy attitudes as well as to uptake cancer prevention interventions such as vaccination and cancer screening [6]. With the aim to increase European Union (EU) citizens’ awareness on cancer prevention, the European Code Against Cancer (ECAC) was set by the European Commission (EC) in 1987 and it has been updated several times. The ECAC is a key educational cancer prevention tool that translates the latest scientific evidence on multiple risk factors and effective interventions, into a list of recommendations for the public to reduce cancer incidence and mortality in the EU. Currently, the ECAC 4^th^ edition is in force, listing "12 ways to reduce your cancer risk" [7], and it is complemented with a website containing extensive information on cancer prevention for non-specialized audiences, in a "question and answer" format [8]. In 2021, a new update of the ECAC was announced by the EC in the Europe’s Beating Cancer Plan, along with a project to develop an EU Mobile App for Cancer Prevention to encourage the uptake of the ECAC recommendations [9].

Periodic population-based surveys conducted in some European countries have studied public’s knowledge of cancer risk factors and symptoms, behaviours, and risk perceptions. However, current literature suggests that knowledge on the association of cancer and risk behaviours such as unhealthy diet or alcohol consumption, is still scarce in the general population [10–12]. In studies recently conducted in Spain, authors concluded that the ECAC lacks real impact as it is unknown to a large part of the population (86.7%; IC95%:85.2–88.2) [13], perhaps because the channels for its dissemination were insufficient and unsuitable for the target population. Addressing this lack of awareness is crucial to empowering the public to make informed choices to reduce the risk of developing cancer and prevent death. Yet, for an educational and health promotion tool to be effective in increasing awareness and to have a significant public health impact, it needs to ensure that a large proportion of the target population is reached and exposed to such educational content [14].

Mobile phones have become the most accessible form of communication in history as they offer mobility, flexibility, convenience, and real-time communication. Currently, there are more than eight and a half billion mobile phone subscribers in the world [15]. Mobile health or “mHealth” refers to the application of mobile technologies, including phones, tablets, etc., as a strategy for supporting and improving the performance of healthcare and public health practices, as it can reduce cost and travelling time for patients and care providers. Given the global reach of mobile phones to a variety of audiences, interventions to deliver cancer prevention education based on mHealth have the potential to impact large groups of people, especially those that are difficult to reach, to produce changes in unhealthy behaviours and improvements in health outcomes. Recent literature examining the effectiveness of mHealth interventions across health topics, such as physical activity, diabetes management or antiretroviral therapy adherence, has showed that these interventions are significantly more effective at improving health outcomes than comparison interventions [16]. However, mHealth applications’ implementation is difficult and complex, involving more challenges in management, social, cultural, and organisational issues than on technical aspects. A variety of factors can influence the effectiveness of mHealth interventions ranging from participant demographics (e.g., gender, age), disease status (e.g., disease self-management, disease prevention), or perceived ease of use, usefulness, security and privacy, and reliability factors influencing adoption of mobile devices [17–20].

With the aim to gather knowledge on the feasibility of a future cancer prevention education intervention, we conducted an implementation research pilot study to assess the *adoption*, *fidelity*, *appropriateness,* and *acceptability* of an intervention to effectively disseminate cancer prevention messages to the public through a mHealth implementation strategy. The study was carried out in the Autonomous Community of Andalusia, the largest in number of inhabitants in Spain (17.9%), which has already implemented a population-based mHealth multi-channel platform to provide healthcare services and personalized health information to its citizens [21, 22].

## Methods

### Study Design

A type-2 hybrid effectiveness-implementation study was designed to primarily test the impact of a mHealth implementation strategy on implementation outcomes (e.g., *adoption*, *fidelity* of intervention delivery) while simultaneously gathering information on the effectiveness of the intervention [23]. In order to simultaneous test both, the clinical intervention and an implementation intervention/strategy, a feasibility single-blind randomised controlled trial was carried out on a sample of the general population in the Autonomous Community of Andalusia, the most populated region of Spain, with more than 8.5 million residents (17.9%) [21, 22]. The Andalusian Health Service (SAS, acronym in Spanish) is the main healthcare provider of the Andalusian Public Health System (SSPA, acronym in Spanish), part of the decentralised Spanish National Health System, which delivers free universal health insurance. The SAS oversees “Salud Responde” (SR, acronym in Spanish), a multi-channel platform that provides a broad online portfolio of services to respond to health-related issues and administrative management needs of citizens and health professionals, complementing the care provided by the healthcare units. It allows 24/7 access to the services and benefits of the SSPA from any point in Andalusia, through telephone, e-mail, short message system (SMS) and a mobile application (app), to manage appointments and reminders, and to disseminate personalized health information [24]. The app has a high coverage with more than 5.7 million users (67% of the Andalusian’s population) registered in its system in 2023 [24]; yet the SR platform maintains the information system service via SMS for specific campaigns to those users who do not have or want the app installed on their mobile phone (around 9700 registered users).

**Fig. 1:**
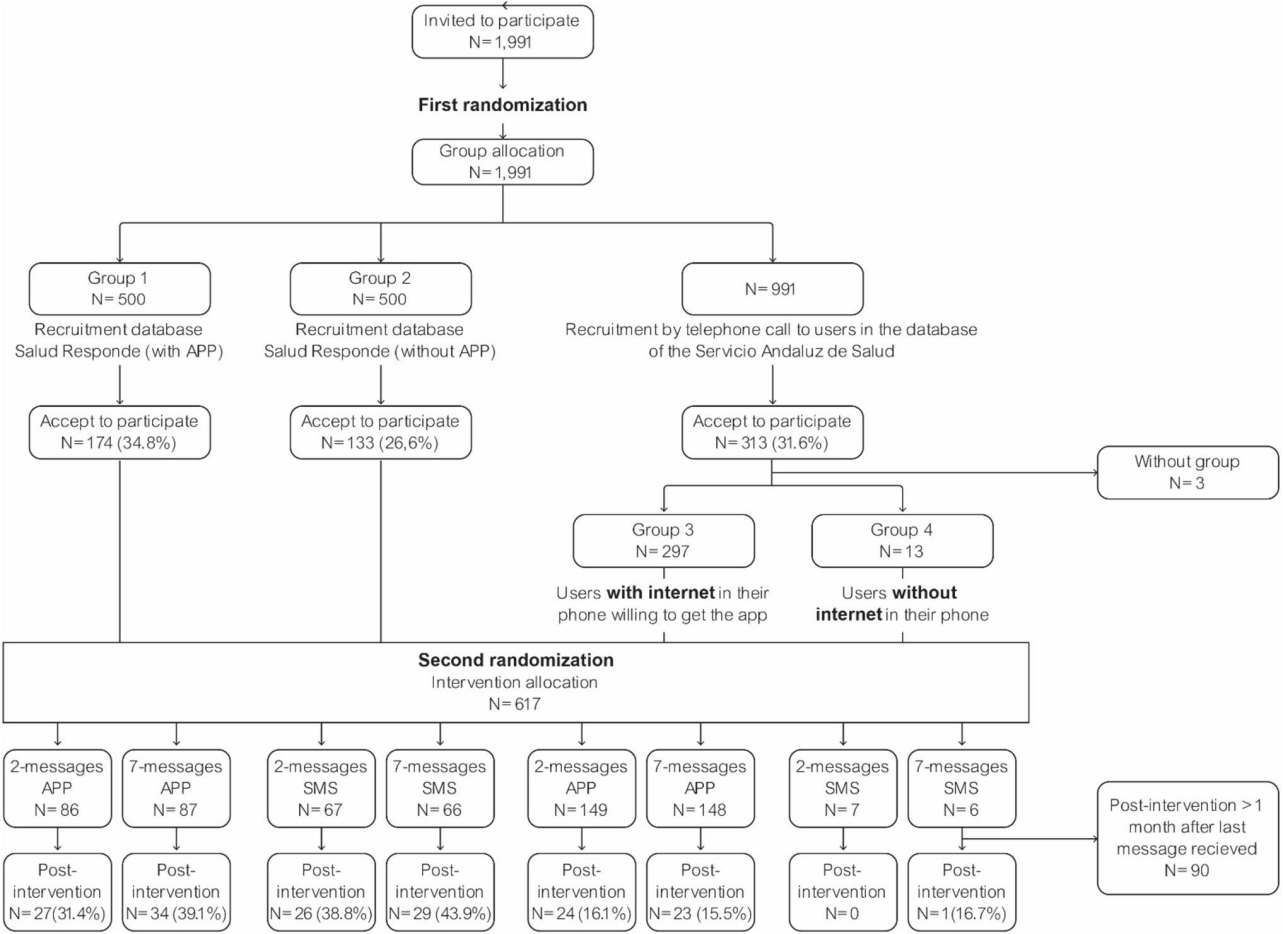
Flowchart describing the two randomizations to allocate the total sample of 1,991 subjects in several groups

The inclusion criteria were being 18 years or over, being entitled to healthcare benefits in SSPA, having a mobile phone, and being able to read messages on them. A random sample of 1991 users from the User Database (BDU, acronym in Spanish) of the SSPA, that in 2019 included more than 8.5 million users [25], was used to assign subjects to the different groups. The first randomization was performed according to the further criteria of being registered or not in the SR platform. Those subjects registered in the SR service platform through an app were assigned to Group 1 (G1), and those registered in the SR service platform through SMS were assigned to Group 2 (G2). The rest of subjects needed to reach the target sample size were recruited from those included in the BDU database but not registered in the SR platform (Fig. 1). For those not registered in the SR platform who accepted to participate in the study, a further distribution was done based on a discriminatory question: those users with internet connection in their mobile phone both through 3G/4G and Wi-Fi were allocated in Group 3 (G3) and encouraged to download the app to receive messages, and those without internet were allocated in Group 4 (G4) to receive messages by SMS. Three subjects did not answer to the discriminatory question.

All participants were recruited through phone call. A maximum of eight attempts were done before considering no response. To be able to calculate participation rate (positive response rate + acceptance to participate), no replacement was performed.

Informed consent to participate was requested and the voluntary and anonymous nature of the participation was ensured. The simple randomisation to the different study groups was performed using the EPIDAT 4.0 programme. The second randomization, explained below in Fig. 1, was performed based on the messages’ delivery frequency: a low frequency of 2 SMS or 2 app notifications (“push”) per week, or a high frequency of 7 SMS or 7 app notifications per week.

### Intervention

After the second randomisation, an intervention was conducted for 4 weeks (between 11 October and 7 November 2021) where participants were sent app “push” notifications or SMS messages via the SR platform. Each “push” notification or SMS message included a cancer prevention recommendation from the ECAC 4^th^ edition (in Spanish) from the following list: "Don't smoke. Do not use any type of tobacco"; "Maintain a healthy weight"; "Exercise daily. Limit time spent sitting"; “Eat lots of whole grains, legumes, fruits and vegetables"; “Limit high-calorie foods (foods rich in sugar or fat) and avoid sugary drinks"; "Avoid processed meat; limit consumption of red meat and salt-rich foods"; “Limit alcohol consumption, although it is best for cancer prevention to avoid alcoholic beverages"; and an URL link to visit the Spanish version of the ECAC’s website to consult additional information [8] through the mobile phone or other digital device. All messages were endorsed and signed by the Head of the Andalusian Regional Ministry of Health. Each participant will receive all 7 messages during the intervention in different frequencies or repetitions.

Data collection was conducted at two time points: at baseline during the recruitment call, a pre-intervention survey was performed until 7 October 2021, to gather basic socio-demographic and patterns of use of new technologies (e.g., type of mobile device, internet access from phone or computer, etc.); and at the end of the intervention, from one week to one month after receiving the last message, a post-intervention survey was carried out by telephone to all participants.

### Variables and Categorization

The sample was described according to the following socio-demographic variables: sex, age, employment status, level of education, living alone or not, and province of residence. The mHealth-related variables included were the type of mobile phone, access to the Internet through the mobile phone or other digital device, permanent access to Internet data or only through Wi-Fi, ability to browse on the Internet (10-point Likert scale, 0 being “no ability” and 10 “outstanding ability”) and from which type of device, and willingness to receive messages in the future (*acceptability* of a future intervention). The survey questions were structured to estimate intervention-related variables: the *adoption of the innovation* (number of messages read), the *fidelity* to the mHealth implementation strategy (receiving and reading the number of messages expected), the preferred frequency and time to receive messages in a future intervention study, willingness to seek further information (number of visits to the ECAC website), and perceived utility and understandability of the messages. Most variables were analysed categorically, except age, ability to browse and the total number of messages read that were continuous.

### Statistical Analysis

The sample size was calculated considering a confidence level of 95% (5% alpha error), a precision of 5% (width of the confidence interval), an expected proportion of 50% (maximum indeterminacy) in the main variables from the Conceptual Framework for Implementation Outcomes (*adoption*, *fidelity*, *appropriateness*, and *acceptability* [23]), and a non-response rate of 5%.

Participation rate was considered an outcome and therefore, calculated as the rate of those who accepted participate in the study among all those who responded to the recruitment call. A descriptive analysis was performed to assess homogeneity across groups by sociodemographic data by means of relative frequencies, using Chi-square test for categorical covariates and *t*-tests for continuous covariates, in the univariate analysis. The sample was also described at follow-up, according to socio-demographic data and patterns of mHealth use by messaging channel. The implementation outcomes of *adoption*, *fidelity*, *appropriateness*, and *acceptability*, and willingness to visit the ECAC’s website (coded as a form of *adoption*), were calculated with relative frequencies by dissemination channel and intervention group. Finally, a multivariate logistic regression was conducted to characterize the participant socio-demographic characteristics associated with having read at least one message weekly adjusting for messages’ delivery frequency. Crude and adjusted Odds Ratios (cOR and aOR) and its 95% confidence intervals (95% CI) were calculated. Significant association was defined with *p* ≤ 0.05. STATA 17.0 and SPSS V.21.0. were used in the analysis.

## Results

Beneficiaries of the SSPA from a sample of 1991, registered or not in the SR platform, were randomly allocated to receive 2 or 7 cancer prevention messages per week through an app notification (“push”) or SMS. To investigate the feasibility of a future large-scale educational intervention at population level, participation rates are important to be estimated as the result of getting a positive response at the recruitment point plus accepting to participate in the study (in other words, the willingness to receive messages among respondents). The total response rate was 67.8% (95% CI 65.7 to 69.8%), however, the percentage fell to 31.0% (95% CI 29.0 to 33.1%) when looking at the participation rate. In the randomized groups, the response rate and participation rates were, respectively: 72.0% (95% CI 67.9 to 75.8%) and 34.8% (95% CI 30.8 to 39.1%) in those using the SR app (G1), and 61.0% (95% CI 56.7 to 65.2%) and 26.6% (95% CI 22.9 to 30.6%) in those registered in the SR platform through SMS (G2); those not registered in the SR platform (G3 and G4 combined) had a response rate of 69.0% (95% CI 66.1 to 71.8%) and participation rate of 31.6% (95% CI 28.8 to 34.5%).

The average age of those who accepted participate in the study was 49 years old, 56.2% were women, 61.9% were employed, 80.4% had secondary education or more, and did not live alone (91.9%). After the second randomization, the distribution of participants across the four groups was homogeneous by sociodemographic variables, except for age, employment status and level of education. The mean age was significantly higher among individuals in G4. This group also had a significantly higher proportion of individuals who were retired and with a level of education of primary or less. The random selection resulted in beneficiaries from the 8 administrative provinces of Andalusia, being Sevilla and Malaga the most populated ones, thus obtaining a geographic representation (Table 1).

**Table 1:** Sociodemographic variables of the sample stratified by groups pre-intervention

			**G1**		**G2**		**G3**		**G4**		
		**Total**	n	%	n	%	n	%	n	%	*p*-value
	**Total**	617	174	28.2	133	21.6	297	48.1	13	2.1	
Sociodemographic variables											
**Sex**	Male	270	69	25.6	60	22.2	135	50.0	6	2.2	0.644
	Female	347	105	30.3	73	21.0	162	46.7	7	2.0	
Age (years)*	*(mean, sd)*	49 (14)	49 (13)		51 (13)		47 (14)		66 (9)		**< 0.001**
**Employment status**	Employed	382	107	28.0	81	21.2	191	50.0	3	0.8	**< 0.001**
	Unemployed	108	32	29.6	27	25.0	48	44.4	1	0.9	
	Retired	103	28	27.2	23	22.3	43	41.7	9	8.7	
	Student	24	7	29.2	2	8.3	15	62.5	0	0.0	
**Level of education**	Primary or less	112	39	34.8	21	18.8	46	41.1	6	5.4	**0.002**
	Secondary	209	60	28.7	37	17.7	109	52.2	3	1.4	
	Technical studies	80	26	32.5	13	16.3	41	51.2	0	0.0	
	University	207	47	22.7	59	28.5	100	48.3	1	0.5	
**Lives alone**	Yes	50	12	24.0	12	24.0	26	52.0	0	0.0	0.614
	No	567	162	28.6	121	21.3	271	47.8	13	2.3	
**Province**	Almeria	45	8	17.8	15	33.3	20	44.4	2	4.4	0.078
	Cadiz	57	19	33.3	5	8.8	30	52.6	3	5.3	
	Cordoba	60	26	43.3	11	18.3	23	38.3	0	0	
	Granada	38	7	18.4	11	28.9	19	50.0	1	2.6	
	Huelva	76	17	22.4	19	25.0	37	48.7	3	3.9	
	Jaen	54	14	25.9	13	24.1	26	48.1	1	1.9	
	Malaga	145	46	31.7	25	17.2	72	49.7	2	1.4	
	Sevilla	141	37	26.2	34	24.1	69	48.9	1	0.7	

Items in bold indicate statistical significance

Chi-squared (*p* < 0.05)

*ANOVA (*p* < 0.05)

At follow-up after the four weeks of intervention, 453 (73.4%) individuals were lost (Table 2). Most common reasons for not answering the post-intervention survey were “not being reachable” (51.7%) and “not willing to participate any longer in the study” (17.8%). In addition, due to the Covid-19 pandemic some disruptions hampered conducting the post-intervention survey at the time planned, therefore, to avoid recall bias, those individuals who answered the post-intervention survey more than one month after they received the last message (N = 90, 19.9%) were excluded from the study and, thus, considered as “lost at follow-up”. Consequently, due to the small sample size at follow-up, the study team decided to merge all subjects receiving the messages through the same channel: G1 and G3 receiving messages through the app, and G2 and G4 receiving messages through SMS. A total sample of 108 (65.9%) participants in the app arm and 56 (34.1%) in the SMS arm completed the post-intervention survey. As shown in Table 2, the group distribution according to reception channel was homogeneous, since no differences were observed by socio-demographic variables nor by mHealth variables, except for age, as individuals receiving the messages through SMS were significantly older than those using the app. As regards mHealth-related variables (Table 2), almost all participants had a smartphone and permanent access to the Internet through it. As for general access to the Internet through other devices, 97 (89.8%) participants from the app group and 44 (78.6%) participants from the SMS group reported being able to access the Internet from a portable computer (PC) or tablet, mostly from home. From a scale from 0 to 10, the mean ability to browse a website of our sample was 7 points. Eighty-eight (81.5%) participants from the app group and 39 (69.6%) from the SMS group felt confident navigating through a telephone (app group), a PC, or a tablet, instinctively. Finally, 91 (84.26%) participants using the app would be willing to receive cancer prevention messages through their phone, in comparison to (78.57%) participants receiving SMS.

**Table 2:** Sociodemographic and mHealth-related variables of the sample stratified by groups post-intervention

			**app (G1 + G3)**		**SMS (G2 + G4)**		
		**Total**	n	%	n	%	*p*-value
**Baseline**		617	471	76.3%	146	23.7%	
**Lost-at follow up**		453	362	79.9%	91	20.1%	
**Post-intervention participants**		164	108	65.9%	56	34.1%	
**Intervention**	2-messages	79	53	0.5	26	46.4%	0.748
	7-messages	85	55	0.5	30	53.6%	
**Sociodemographic**							
**Sex**	Male	77	52	48.1%	25	44.6%	0.704
	Female	85	55	50.9%	31	55.4%	
Age (years)*	*(mean, sd)*	49 (12)	47 (12)		52 (12)		**0.009**
**Employment status**	Employed	106	76	70.4%	30	53.6%	0.108
	Student	28	15	13.9%	13	23.2%	
	Unemployed	22	11	10.2%	11	19.6%	
	Retired	4	3	2.8%	1	1.8%	
**Level of education**	Primary or less	30	18	16.7%	12	21.4%	0.655
	Secondary	43	28	25.9%	15	26.8%	
	Technical studies	18	14	13.0%	4	7.1%	
	University	71	46	42.6%	25	44.6%	
**Lives alone**	Yes	15	11	10.2%	4	7.1%	0.510
	No	148	96	88.9%	52	92.9%	
**mHealth-related variables**							
**Type of mobile telephone**	Traditional	7	3	2.8%	4	7.1%	0.194
	Smartphone	156	104	96.3%	52	92.9%	
**Access to Internet from mobile phone**	Only with Wi-Fi	6	2	1.9%	4	7.1%	0.089
	Permanent	157	105	97.2%	52	92.9%	
Ability to browse*	*(mean, sd)*	7 (2)	8 (2)		7 (3)		0.119
**Willingness to receive messages**^**a**^	Not very	3	1	0.9%	2	3.6%	0.409
	I don't care	14	9	8.3%	5	8.9%	
	Quite	67	42	38.9%	25	44.6%	
	Very	68	49	45.4%	19	33.9%	

Items in bold indicate statistical significance

Chi-squared (*p* < 0.05)

^*^t-student (i < 0.05)

^a^12 missing

### Assessment of Implementation Outcomes Related to the Intervention

As defined by Proctor et al*.* [26], “*adoption or uptake* is the intention, initial decision, or action to try or employ an innovation or evidence-based practice’’. In our study, *adoption of the innovation* was defined as the number of messages read, and 80.3% of all participants reported have read “all” or “most of” the total messages sent, regardless of the dissemination channel and the frequency. Yet, to avoid recall bias at the end of the intervention, *adoption* was also assessed as “having read at least one message weekly” and we found that, in the group of individuals who answered the post-intervention survey, 143 (87.2%) reported so (Table 3). When looking at the dissemination channel and the frequency of the messages, 26 (100%) of participants receiving 2 SMS per week and 27 (90%) receiving 7 SMS per week read at least one message weekly, whereas 40 (75.5%) of the participants receiving 2 “pushes” through the app per week and 50 (90.9%) of the participants receiving 7 “pushes” per week read at least one message weekly. In comparison, participants receiving SMS were reading a greater number of weekly messages than those receiving them through the app; however, those receiving 7 messages per week, either by SMS or app “push”, were reading less than three messages per week. When we conducted an adjusted logistic regression to understand the predictors of *adoption*, we observed that those with a university degree were three time more likely to report having read at least one message weekly (aOR = 3.02; CI 95% 1.11-8.23) compared to those with primary education or less.

**Table 3:** Adoption and fidelity of the intervention in total post-intervention participants

					**Adoption**				**Fidelity**			
	**Baseline participants** **(pre-intervention)**		**Total participants (post-intervention)**		**At least one message read weekly**^a^		**Mean number of weekly messages read**^a^		**Received the expected number of messages weekly**^a^		**Read the expected number of messages weekly**^a^	
**Study arm**	n	%	n	%	n	%	mean	sd	n	%	n	%
**Total**	**617**	**100.0%**	**164**	**26.6%**	**143**	**87.2%**	**2.52**	**0.21**	**23**	**14.0%**	**24**	**14.6%**
2-SMS	74	12.0%	26	15.9%	26	100.0%	2.12	0.27	9	34.6%	11	42.3%
7-SMS	72	11.7%	30	18.3%	27	90.0%	2.97	0.45	0		0	
2-app	234	37.9%	53	32.3%	40	75.5%	1.40	0.29	13	24.5%	12	22.6%
7-app	236	38.2%	55	33.5%	50	90.9%	2.27	0.38	1	1.8%	1	1.8%

Items in bold indicate statistical significance

^a^As declared by the participants

As regards the implementation research outcome of *fidelity*, which is “the degree to which an intervention was implemented as it was prescribed in the original protocol or as it was intended by the program developers” [26], all groups declared a total number of messages received and read weekly lower than expected, as only 23 participants (14.0%) reported having received and 24 participants (14.6%) having read the expected number of messages. Almost all of them were allocated to the frequency of 2 SMS or 2 app “pushes” per week in the second randomization.

*Appropriateness* or “the perceived fit, relevance, or compatibility of the innovation or evidence-based practice for a given practice setting, provider, or consumer” [26], was measured through the usefulness or utility, and understandability of the messages, as well as preferences regarding the time and the frequency to receive these messages. Most participants found the messages useful, understandable, timely, and appropriate in their weekly frequency (Table 4). However, participants receiving messages through SMS reported higher *appropriateness* shown by a higher understandability (*p* = 0.010) and more adequate frequency (*p* = 0.015) than those receiving messages via “push” notification of the app. *Appropriateness* rates were homogenous among those who received 2- and 7-messages both via app and SMS for all analysed criteria, except for usefulness, as the proportion of participants finding messages useful was significantly lower among the 7-SMS arm (*p* = 0.029).

**Table 4:** Appropriateness, acceptability, and adoption of the intervention in total post-intervention participants

				**app (G1 + G3)**		**SMS (G2 + G4)**		
				n	%	n	%	
			**Total**					*p*-value
		**Total**	164	108	65.9%	56	34.1%	
**Appropriateness**	**Usefulness/Utility**	No	27	22	20.4%	5	1.8%	0.061
		Yes	137	86	79.6%	51	42.9%	
	**Understandability**	No	12	12	11.1%	0	0.0%	**0.010**
		Yes	152	96	88.9%	56	100.0%	
	**Timing**	No	4	2	1.9%	2	3.6%	0.051
		Yes	118	72	66.7%	46	82.1%	
		DK^a^	42	34	31.5%	8	14.3%	
	**Frequency**^b^	No	49	39	36.1%	10	17.9%	**0.015**
		Yes	115	69	63.9%	46	82.1%	
**Acceptability**	**Willingness to receive more messages (acceptability of a future intervention)**	No	24	19	17.6%	5	8.9%	0.137
		Yes	140	89	82.4%	51	91.1%	
**Adoption**	**Access to complementary information**	No	130	85	78.7%	45	80.4%	0.804
		Yes	43	23	21.3%	11	19.6%	

Items in bold indicate statistical significance

Chi-squared (*p* < 0.05)

^a^Don’t know

^b^stratified analysis by intervention group was homogeneous in those receiving 2 and 7 messages through both, the app and the SMS, therefore, global data are presented as for the rest of the variables

*Acceptability* is an implementation research outcome that is key for the success of the implementation of an intervention, practice, technology, or service within a particular setting of care. It is defined as “the perception among implementation stakeholders that a given treatment, service, practice, or innovation is agreeable, palatable, or satisfactory” [26]. To capture this construct in our study, we measured participants’ willingness to receive more messages in the future, through a scaled-up mHealth cancer prevention education intervention. Similarly, to the high *appropriateness* of the intervention according to the four criteria described above, participants in both arms of the intervention that completed the study showed a high *acceptability* to receive more messages in the future (82.4% though the app and 91.1% through SMS). No significant differences were observed between arms.

Finally, we assessed participants’ access to complementary information (which, for the purpose of this study, was also defined as *adoption*); and, despite the high *acceptability* rates reported to participate in a future mHealth intervention with similar characteristics, only 43 (26.2%) participants visited the website of the ECAC 4^th^ edition to seek further information, as encouraged in the text messages sent. The most frequent reasons for not accessing the website were “lack of time” (22.0%) and “not being interested” (11.6%).

## Discussion

Implementation research aims to close the research-to-practice gap, and support scale-up of evidence-based interventions. It involves studying the challenges and strategies involved in deploying new innovations and technologies in real-world healthcare settings, by measuring implementation outcomes, which are distinct from patient outcomes (e.g., symptoms, behaviours) and health service outcomes (e.g., efficiency, safety). Proctor et al. proposed a taxonomy of eight implementation outcomes, defined as “the effects of deliberate and purposive actions to implement new treatments, practices, and services” consisting of: *acceptability*, *appropriateness*, *adoption*, *feasibility*, *fidelity*, *implementation cost*, *penetration*, and *sustainability* of evidence-based practice [26]. In our study, we aimed to measure some of these outcomes as indicators of the feasibility of deploying a cancer education intervention using mHealth as implementation strategy, through the currently in use SR platform of the SAS that covers 67% of the Andalusian’s population.

Although mHealth is a powerful implementation strategy, still most of the studies focus on the efficacy of interventions to improve complex lifestyle behaviours in the short term, instead of the impact at population level. Some studies assessing the effectiveness (most frequently through SMS-based interventions) have provided preliminary evidence of small, sustained effects to produce positive change in preventive behaviours [27]; positive results in chronic disease management, reduction of deaths and hospitalisation; or improvement of healthcare attendance rates; however, the methodological quality of most studies has been described as low [28]. Well-designed population-based implementation research studies are needed to investigate the external validity or “real-world” impact of a scaled-up intervention to reach massive dissemination effectively, and to gather information on the coverage, acceptability, and adherence to the intervention, as most mHealth research shows high self-reported acceptability to receive messages but low coverage or reach of the intervention [29]. In line with this, our results show an overall low participation rate in comparison to more than double initial positive response rate, showing that there is a considerable mismatch between the theoretical coverage that the intervention could have achieved and what happened in practice. Our study also shows generally high access to the Internet through the mobile phone or other devices, high ability and confidence to browse a website, and high willingness to receive cancer prevention messages in the phone. However, while the uptake or *adoption* was shown to be high in those participants that completed the study, in particularly in the SMS arm, a discrepancy with the results on the *fidelity* of the intervention to the implementation plans becomes apparent, as messages received and read weekly, either by app or SMS, were lower than expected. Recall bias may explain at least part of these results [30], along with social desirability, which is the tendency of respondents to distort self-reports by providing responses that they think are consistent with social norms and expectations [31]. In addition, data show a high recall bias in the higher frequency group, receiving 7 “push” notifications or 7 SMS per week, as most of them recalled having received or read a smaller number of messages than anticipated; and only one reported having received and read seven messages over the last week. In the app group, this may be explained by the fact that the “push” notification needed to be activated by the user to acknowledge reception of the messages; also, some users may have uninstalled the app during the study, without notifying the interviewers at the post-intervention survey. To overcome this, system usage data should be enhanced to quantitatively measure engagement with mHealth interventions [32].

As regards *appropriateness* of the implementation strategy, receiving cancer prevention messages through mHealth was shown to be appropriate in terms of the frequency and the time when the messages were received. Likewise, the messages were well understood, especially by those in the SMS group, and perceived useful, mainly by those receiving 2 SMS per week. However, the willingness to use complementary communication modalities such as website as a source of additional information, was shown to be low (below 22% in both groups). Finally, when assessing the *acceptability* or potential success of scaling-up the intervention, and despite the inconsistency with the fidelity outcome, the willingness to receive more messages in the future was shown to be very high (above 80% in the app group and 90% in the SMS group).

### Implications for mHealth Use in Cancer Prevention

The Europe’s Beating Cancer Plan is deploying an ambitious plan to tackle cancer all along its pathway, putting a special emphasis on prevention, and aiming to disseminate cancer prevention through mHealth boosting the usability of a future EU Mobile App for Cancer Prevention [9, 33]. The ECAC regularly gathers the most up to date evidence-based actions and interventions on cancer prevention, with the aim of translating this knowledge to the public and influence their behaviour to reduce the cancer burden in the EU in the long run. Although some awareness on cancer prevention and the ECAC exists in certain EU countries, it is not yet known to what extent it will be able to bring about changes in knowledge, attitudes, and behaviours towards cancer prevention in a sustained and long-lasting way [34]. Indeed, less than 3% of participants in the last French Cancer Barometer reported knowledge on established cancer risk factors such as lack of physical activity, sun exposure, or overweight [10]. Similarly, the last Spanish National Onco-barometer showed that knowledge of cancer risk factors and symptoms was limited among males and older individuals [12]. Findings from the recent National Survey on Cancer Awareness and Attitudes from Ireland also showed lower levels of recognition of cancer risk associated with alcohol consumption, dietary factors, physical activity, body weight or breast feeding, in comparison to tobacco or sun exposure [11]. To increase cancer prevention awareness to foster behavioural change, mHealth has the potential to reach large numbers of people across the EU through more efficient and targeted healthcare. However, large uptake and effective user’s engagement needs to be accounted for [35], as well as inequalities in digital health, as the literature reports that White, English-speaking, young, highly educated, of higher economic status, and residents in urban areas, are the more prominent users of digital health technologies [36]. According to this, most of participants in our study were middle aged, employed and with secondary education or more. Although the study did not intend to calculate statistical differences between dissemination channels, it was observed that older, retired, and with a lower level of education people continue to use SMS against newer technologies. These data would need to be considered to effectively reach certain groups of society that are generally harder to reach.

### Limitations and Disruptions on Fidelity Caused by the Covid-19 Pandemic

In general, recall bias seems to be a relevant limitation in the study. This highlights the importance of not relying exclusively on self-reported data in future studies and reducing the time-lag between the end of the intervention and the post-intervention data collection. In addition, some of the limitations of the study were due to the disruptions caused by the Covid-19 pandemic that impeded the implementation of the study protocol as it was originally designed. First, the sample of participants was selected in 2019, however, the pre-intervention survey and recruitment call could only be conducted in 2021. This two-year lag could have decreased our participation rate since, during this time, some potential participants could have changed their number or have even died. Second, half of the participants were not reachable to respond to the post-intervention survey (51.7%) and 17.8% were not willing to participate any longer in the study. Although this is only a hypothesis, we believe participants may have experience a sort of fatigue related to receiving so many calls related to the Covid-19 pandemic (e.g., reminders to attend vaccination, of public health measures, etc.). Third, the post-intervention was heavily affected as all SR operators were responding to Covid-19-related calls, therefore, not all the post-intervention calls were made within one-month as established in the protocol. As a result, the study team took two decisions that may have negatively influenced the *fidelity* of the study: 1) to boost the messages through an extra wave for one week (either app or SMS) between 19 and 26^th^ January 2022; and 2) to exclude those participants that responded to the post-intervention survey more than one month after the last message was received, to avoid recall bias, increasing the loss at follow-up number. Finally, due to the resulted small sample size the study team decided to merge groups G1 and G3, and G2 and G4, to perform the analysis by message channel only, as the low participation rates did not allow us to compare groups by recruitment procedure to assess motivation to download the SR app. Other limitations refer to some inconsistencies observed, even though almost all participants reported having a smartphone and permanent access to the Internet. This highlighted some misinterpretations at the time of answering the pre-intervention survey, for example: three participants reported having a traditional phone or two reported not having internet in their phone, while for all five cases the database confirms that they had the app installed in their mobile phones, or one participant from G4 reported having access to the Internet while having a traditional phone.

## Conclusions

The observed data show that an intervention for cancer prevention education, using mHealth as implementation strategy, is acceptable and feasible. However, to investigate potential changes in attitudes and knowledge about cancer prevention as a prerequisite for effective behavioural changes, before scaling-up such an intervention, it would need to be adjusted to increase coverage and *adoption* of the intervention*,* and *fidelity* would need to be ensured, to guarantee penetration and sustainability in the healthcare system of Andalusia. The results of this study may contribute to inform the design of future interventions using mHealth at large scale, such as the future EU Mobile App for Cancer Prevention, to achieve a broad coverage across the EU as well as to ensure *adoption* of the cancer prevention messages as those promoted by the ECAC.

## Data Availability

The datasets generated and/or analysed during the current study are available in the [BASE DE DATOS DE PERSONAS USUARIAS (BDU)] repository, [https://www.sspa.juntadeandalucia.es/servicioandaluzdesalud/archivo-estadisticas/base-de-datos-de-personas-usuarias-bdu-2].

## Abbreviations

app: Mobile application
BDU: User Database (acronym in Spanish)
EU: European Union
ECAC: European Code Against Cancer
EC: European Commission
PC: Portable computer
SAS: Andalusian Health Service (acronym in Spanish)
SSPA: Andalusian Public Health System (acronym in Spanish)
SR: Salud Responde (in Spanish)
SMS: Short message system
